# Analysing the influence of ground granulated blast furnace slag and steel fibre on RC beams flexural behaviour

**DOI:** 10.1038/s41598-024-51164-x

**Published:** 2024-02-28

**Authors:** A. Ramachandra Murthy, P. K. Prasanna, G. Nipun, K. Srinivasu, Kumaraswamy Gandla, Afzal Husain Khan, Ehab Sabi

**Affiliations:** 1 CSIR-Structural Engineering Research Centre, Chennai, 600113 India; 2 Department of Civil Engineering, V R Siddhartha Engineering College, Vijayawada, AP India; 3 NRI Institute of Technology, Guntur, AP India; 4 Department of Pharmaceutical Analysis, Chaitanya University, Hanumakonda, India; 5 Civil Engineering Department, College of Engineering, Jazan University, PO Box 706, 45142 Jazan, Saudi Arabia

**Keywords:** Environmental sciences, Materials science

## Abstract

This study examines the effect of Ground Granulated Blast Furnace Slag** (**GGBS) and steel fibers on the flexural behaviour of RC beams under monotonic loading. Various percentages of GGBS were used to substitute cement, namely 0%, 20%, 40%, 60%, and 80% and fibers were added to the concrete mix as 0%, 0.5%, 1%, and 1.5% of the volume of concrete. The load–deflection behaviour of GGBS-incorporated RC beams with fibers was compared with the control RC beam. Beams were tested under load control for 28 days and 180 days. The ultimate load of the GGBS-incorporated RC beam up to 40% cement replacement was found to higher than that of the control beam. The strength of concrete is reduced by 28% and 19% when cement was partially replaced by 80% of GGBS at 28 and 180 days, respectively, compared to control concrete without fibres. Further, the analytical load–deflection response of GGBS-incorporated RC beams was determined by using several codes of practice, namely, ACI 318-11(2011), CSA A23.3-04 (2004), EC-04 (2004), and IS 456 (2000). The Codal provisions were primarily based on the effective moment of inertia, Young’s modulus, and modulus of rupture, stiffness, and cracking. Average load–deflection plots obtained from experiments were compared with the computed load–deflection of analytical studies. It was found that the analytically predicted load–deflection behaviour is comparable with the corresponding average experimental load–deflection response. Moment curvature relations were also developed for RC beams.

## Introduction

Concrete is extensively used when building civil infrastructure like bridges, airports, buildings, power plant structures, etc. In order to generate the necessary concrete, there will be an enormous increase in the demand for cement, which will result in higher CO_2_ emissions. Different supplementary cementitious materials are being used in the concrete in place of some of the cement, which will help the greenhouse effect. In the current investigation, GGBS substitutes cement in varying amounts. Ground Granulated Blast Furnace Slag** (**GGBS), a by-product of the iron industry produced by quenching molten slag, has cement's chemical composition. About 530 million metric tonnes of GGBS are produced worldwide each year, of which the construction sector uses 65%. The addition of GGBS to concrete results in (i) enhanced strength and durability of concrete structure (ii) better workability (iii) less thermal and shrinkage cracks (iv) more flexural strength etc. Fibers are frequently used in concrete to enhance its ductility, toughness, fatigue, impact resistance, fracture bridging, and other qualities.

In the current investigation, steel fibers have been used. Reinforced Concrete (RC) beams with longitudinal and shear reinforcement composed of M60 concrete grade has evaluated for their flexural behaviour under cyclic loading. The experimental findings revealed an increment in strength, deformation, and ductility properties and a decrease in crack frequency and width^1^. The beam strength was measured under three-point bending. It has been prepared with M20-grade concrete and reinforced with twisted steel fibers. Steel fibers were proven the flexural strength should be increased and the toughness of materials^2^. The characteristics of concrete have been determined when the beams made with GGBS are replaced at percentages of 0%, 50%, 70%, and 90%. Because of its performance, GGBS concrete RCC beams have demonstrated that GGBS up to 70% may be utilized without sacrificing the stiffness and strength of the RCC beams^3^. Under cyclic loading, the RC beams with various percentages of steel fibers have performed. The impact of steel fibers on cyclic behaviour has been compared with that of the absence of steel fibers, and several characteristics have been evaluated. It has been revealed that the maximum strength and ultimate changes in RC beam displacement with steel fiber percentages of up to 2% show an enhancement in the cyclic behaviour^4^.

Using Ultra High-Performance Fibre Reinforced Concrete (UHPFRC), reinforced concrete beams’ flexural strength can be predicted, a model was created utilising the parameters elastic modulus and compressive strength, the volume % of steel fibres, as well as the characteristics of the beam's geometry. The reinforced beams exhibit the modes of failure, comprised of concrete cover separation, reinforcement yielding, and UHPFRC layer rupture. The estimated values were compared with the experimental results^5^. Investigations on the mechanical properties, including load–deflection behaviour, fracture development, maximum load capacity, and failure modes, employed hunched beams. The several hunched beam configurations with variable beam depth, width, and reinforcement were also compared^6^. The Digital Image Correlation (DIC) system will capture pictures throughout the entire loading process. Testing was done with controlled loading on the RC beams. DIC software will use the pictures to derive strain and deformation data. They also compared the outcomes of the investigations to those attained by analytical simulations^7^. Durability and long-term performance are crucial in the design of concrete structures, then the steel-fiber- and nano-silica-infused concrete was used. It leads to improved crack resistance, energy absorption capacity, enhanced bond strength, and also improves fracture properties^8^. The material combination, fiber content, and the experimentally established Self Compacting Concrete (SCC) characteristics all greatly impacted the fracture properties of ternary blended fiber reinforced SCC^9^. To detect cracks and failures in concrete structures, image processing techniques and the development of suitable machine-learning algorithms are used. The outcome of the experiment has contrasted with Python programming. RCC beams were cast using basalt and glass fibers for this study, and the deformations and fracture widths were assessed using a four-point static bending test^10^. It was determined how worn-out, GGBS-based UHPC-reinforced beams responded under fatigue. After that, analytical models were used to compare the outcomes. It just takes 5 mm thick GGBS-based Ultra High Performance Concrete (UHPC) strips to increase the reinforced beam’s fatigue life^11^. The combination of 20% pond ash and 30% GGBS produced the most effective mix and was inferred from the experimental findings. A model should also be developed using the ABAQUS programme, and the outcomes should be verified against the experimental results^12^.

To understand the behaviour of the load deflections, perform the flexural tests on repaired geopolymer concrete beams. Then the tested beams were repaired with epoxy resin injections and underwent static loading testing. Experimentally proved the restored beams outperformed the original beams in preventing the development of new cracks^13^. The physical attributes of dry UHPC fiber-concrete have been studied under various curing conditions. The results revealed that there is a change of behaviour following cracking when the volume fraction of fibers is 1.5%^14^. This work evaluated the concrete-reinforced FRP bar-reinforced beam deflections using experimental and analytical methods. There are new formulae for the effective moment of inertia in concrete beams reinforced with FRP bars based on experimental findings using Branson's equation were proposed. The equations have been formulated so that genetic algorithm optimization significantly reduces the discrepancies between predicted values and experimental findings^15^. Evaluate the efficacy of the thermal insulation offered by concrete manufactured with various cementitious composites. Compounds made from barite and pumice has also been demonstrated to be radiation-resistant. Perlite and pumice-impregnated composites show comparatively low heat conductivity^16^. The fatigue strains are minimum in UHPC materials. The fatigue strains in structural elements increased during cyclic loading, and they absorbed the dissipated energy. Determining the long-term performance of strengthened RC structure’s bonding material is also crucial in their design and evaluation^17^.

From the above literature, it can be noted that the behaviour of GGBS-incorporated RC beams with fibers has not been investigated to the best of authors’ knowledge. Hence, in the present study, experimental and analytical investigations were carried out on the flexural behaviour of GGBS-incorporated RC beams with and without fibers. Cement has been replaced by GGBS up to 80%, and fibers were added in proportions of 0.5%, 1.0% and 1.5%.

## Experimental details

### Materials

**Figure 1:**
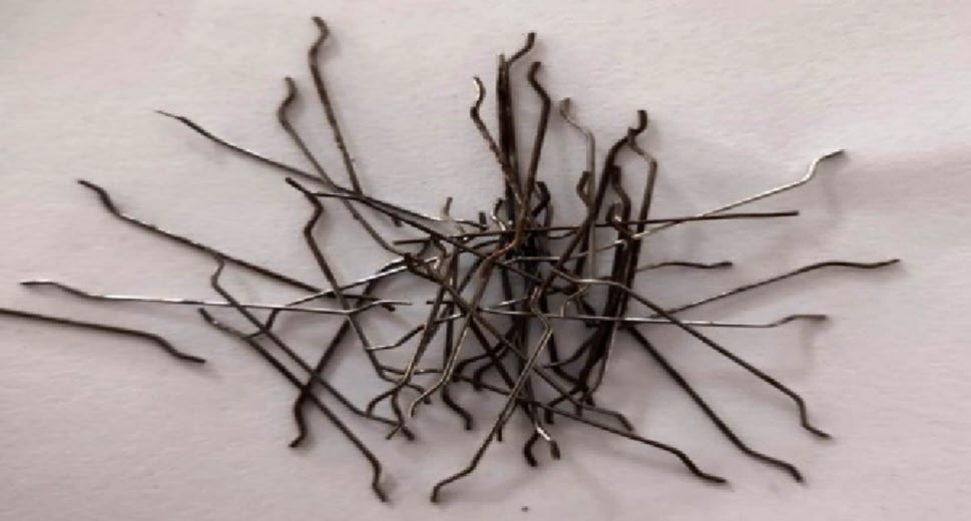
Steel fibers.

The specific gravity of 53 grade Ordinary Portland Cement (OPC) of 3.13 is used in accordance with IS 12269-1987^18^. For this mixture, Sand from rivers conforms to IS: 383-1970^19^ Grading Zone II is employed and sizes 20 mm and 12 Coarse aggregates are utilised in mm. Hooked-end steel fibers are used in concrete mixes incorporated with and without GGBS. In addition to having an atomic weight of 2.88, GGBS has a specific surface area and fineness of 4250 cm^2^/g and 4% respectively. In order to determine the mix proportions, the absolute volume approach was used. The properties of the GGBS as per IS12089-1987^20^, coarse and fine aggregates, and steel fibres are shown in Tables 1, 2, and 3, respectively. The current study, hook-ended steel fibres according to ASTM A 820-01-2001^21^ are used for concrete mixes, as shown in Fig. 1.

**Table 1:** Chemical properties of GGBS.

Parameter	Value (%)	IS: 12,089-1987
Calcium oxide	37.41	–
Silicon dioxide	37.54	–
Aluminum oxide	14.23	–
Iron oxide	1.22	–
Magnesium oxide	8.65	Max. 17%
Sulphide sulphur	0.36	Max. 2%
Loss of ignition	1.34	
Insoluble residue	1.64	Max. 5%
Glass content (%)	91	Min. 85%

**Table 2:** Properties of fine and coarse aggregates.

Parameter	Fine aggregate	Coarse aggregate
Loose bulk density (kg/m^3^)	1510	1495
Compacted bulk density (kg/m^3^)	1620	1715
% of voids	42.47	36.18
Fineness modulus	2.44	7.23
Specific gravity	2.63	2.71
Water absorption (%)	1.13	0.44

**Table 3:** Properties of steel fibers.

Parameter	Value
Length of the fiber (L)	30 mm
Diameter of the fiber (D)	0.5 mm
Aspect ratio (L/D)	60
Tensile strength of fiber	1100 N/mm^2^
Young’s modulus of fiber	2 × 10^5^ N/mm^2^
The density	7800 kg/m^3^

### Geometry and detailing of reinforced cement concrete (RCC) beam

**Figure 2:**
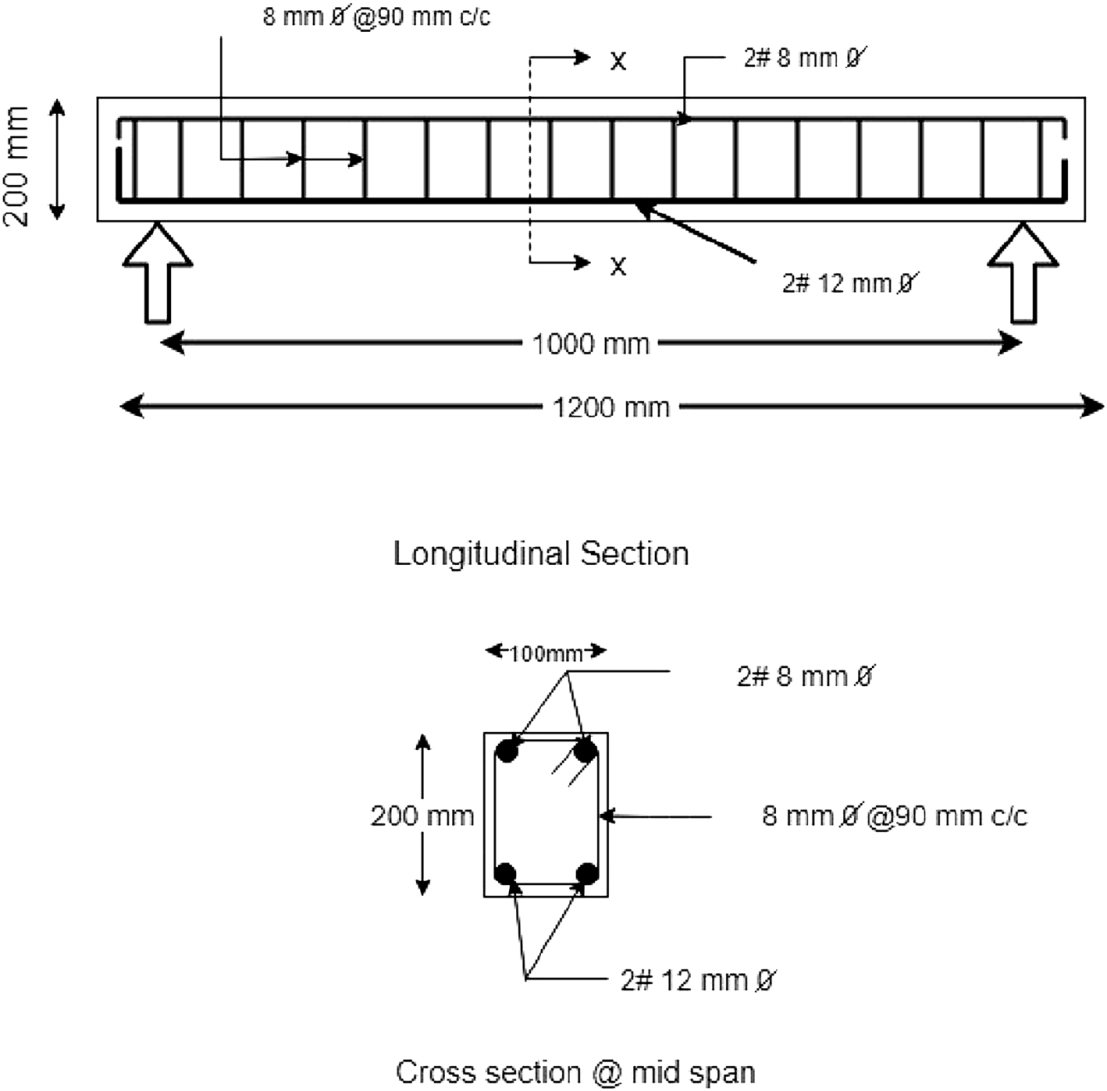
Detailing of RCC beam.

**Figure 3:**
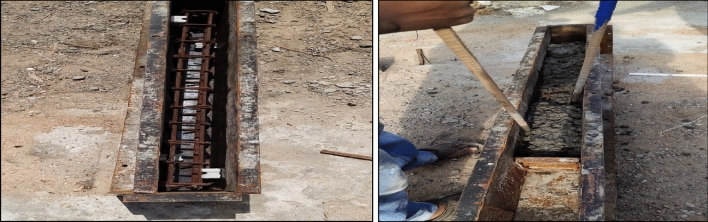
Casting of RCC beam.

The geometry of the RCC beam is 1200 (length) × 100 (width) × 200 (depth) mm. The beams were cast with tension steel, 2 no’s of 12 mm bars, in the compression zone reinforced with anchor bars of 2 no’s of 8 mm diameter bars, and the longitudinal bars tied with 8 mm diameter stirrups spaced at 90 mm c/c. The yield strength of steel (fy) is 500 MPa; the characteristic strength of concrete at the age of 28 days (f_ck_) is 35 MPa. The clear cover provided for reinforcement is 20 mm. The average compressive strength (cubes of 150 × 150 × 150 mm), flexural strength (prisms of 500 × 100 × 100 mm), and split tensile strength test (cylinders of 100 × 200 mm) of concrete at 28 days of strength with 0% GGBS and 0% fibers were 43.83 MPa, 3.68 MPa, and 4.84 MPa, respectively. Conventional concrete beams with a cement content of 450 kg/m^3^ with and without fibers were cast. Figure 2 shows the geometry and cross-section of RC beams, and the typical casting of the beam is shown in Fig. 3. The properties of structural steel are shown in Table 4. The details of the mix proportions of GGBS-based RC beams, water to binder ratio and volume fraction of fibers are specified in Table 5. For each mix/composition, three beams were tested, and the average performance is reported.

**Table 4:** Structural steel properties.

S. No	Diameter (mm)	Area (mm^2^)	Yield stress (N/mm^2^)	Ultimate stress (N/mm^2^)	Percentage elongation
1	8	50.32	553.23	577.82	25.5
2	12	113.25	594.06	656.84	24

**Table 5:** Mix designation and reinforcement details of GGBS-based RC beams with and without fibers.

Mix ID	Mix designation	Reinforcement in beams				The volume of fibers (V_f_) and wt. in kg/m^3^
		Longitudinal bars		Stirrups		
		Top (mm)	Bottom (mm)	Diameter (mm)	Spacing (mm)	
M9	C 450-0.5-G-00%, V_f_-0%	2#8	2#12	8	90	0
M9-1	C 450-0.5-G-20%, V_f_-0%					
M9-2	C 450-0.5-G-40%, V_f_-0%					
M9-3	C 450-0.5-G-60%, V_f_-0%					
M9-4	C 450-0.5-G-80%, V_f_-0%					
M9 A	C 450-0.5-G-00%, V_f_-0.5%	2#8	2#12	8	90	39
M9-1A	C 450-0.5-G-20%, V_f_-0.5%					
M9-2A	C 450-0.5-G-40%, V_f_-0.5%					
M9-3A	C 450-0.5-G-60%, V_f_-0.5%					
M9-4A	C 450-0.5-G-80%, V_f_-0.5%					
M9 B	C 450-0.5-G-00%, V_f_-1%	2#8	2#12	8	90	78
M9-1B	C 450-0.5-G-20%, V_f_-1%					
M9-2B	C 450–0.5-G-40%, V_f_-1%					
M9-3B	C 450-0.5-G-60%, V_f_-1%					
M9-4B	C 450-0.5-G-80%, V_f_-1%					
M9 C	C 450-0.5-G-00%, V_f_-1.5%	2#8	2#12	8	90	117
M9-1C	C 450-0.5-G-20%, V_f_-1.5%					
M9-2C	C 450-0.5-G-40%, V_f_-1.5%					
M9-3C	C 450-0.5-G-60%, V_f_-1.5%					
M9-4C	C 450-0.5-G-80%, V_f_-1.5%					

### Test setup for GGBS-based RC beam

**Figure 4:**
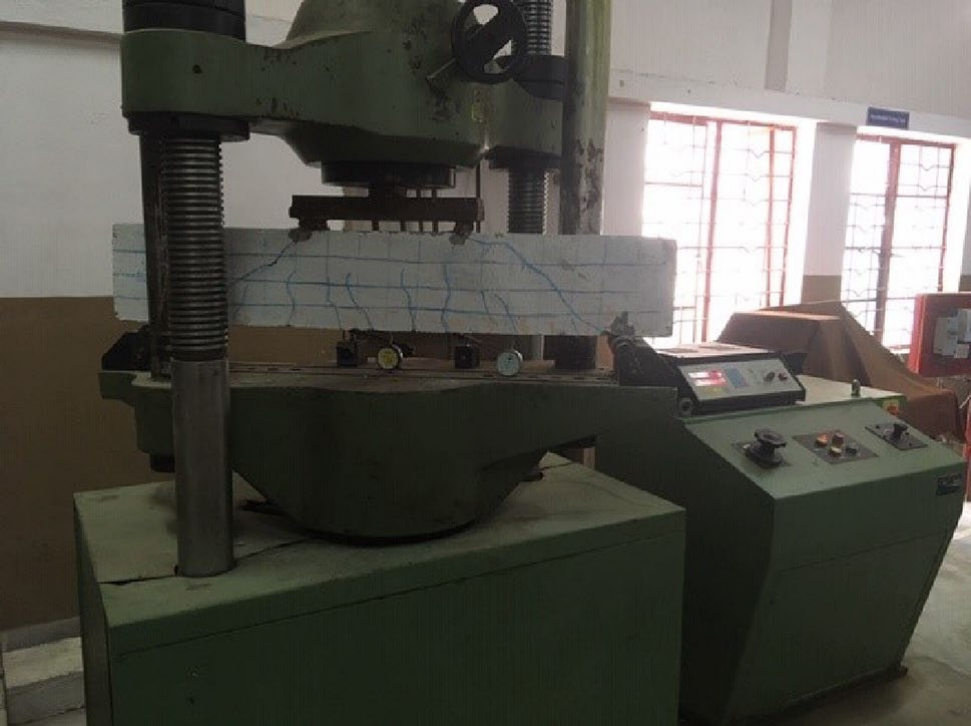
Test setup for RC beam.

Consider a typical simply supported RC beam subjected to a gradually increasing load (Fig. 4). The central portion of the beam is subjected to pure flexure. The beams were tested under load control by applying a two-point load through the universal testing machine having a 1000 kN capacity. The effective span of the beam is 1000 mm, and the load is applied at two points, each 330 mm away from the centre of the beam towards the support. The deflections at the mid-span were measured with the help of a dial gauge, which has the least count of 0.001 mm. The reading of the dial gauge was recorded for different loadings. The crack initiation load, ultimate load, and failure loads were recorded during the testing. During testing, the crack propagation was noticed, and this continued until the test was terminated.

## Results and discussions

### General observations made during the experimental test

**Figure 5:**
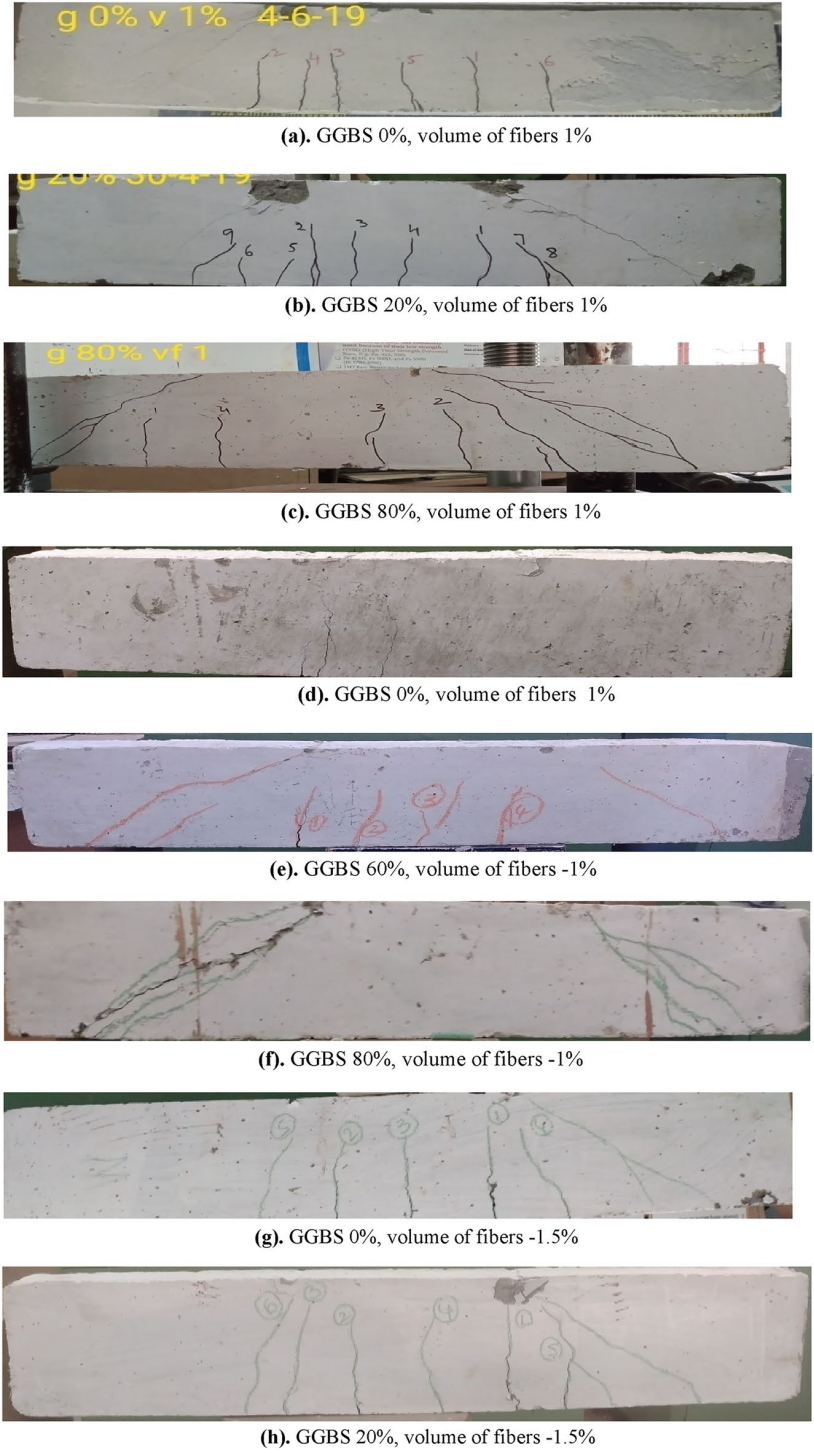
Crack pattern in typical beams. (**a**) GGBS 0%, volume of fibers 1%. (**b**) GGBS 20%, volume of fibers 1%. (**c**) GGBS 80%, volume of fibers 1%. (**d**) GGBS 0%, volume of fibers 1%. (**e**) GGBS 60%, volume of fibers − 1%. (**f**) GGBS 80%, volume of fibers − 1%. (**g**) GGBS 0%, volume of fibers − 1.5%. (**h**) GGBS 20%, volume of fibers − 1.5%

The aim of the experimental test is to assess the flexural behaviour of concrete beams made using GGBS. It consists of conventional RC beams as well as concrete beams made up of GGBS with and without fibres. Initially, flexural cracks (vertical) were observed in the mid-span of the beam in the region of constant bending moment, and ultimate failure occurred due to the crushing of concrete in the compression zone with the maximum deflection. These cracks were observed in all beams. The formation of crack width is within the limits of IS Codal provisions (ranging between 0.15 mm and 0.2 mm) at service loads. The concrete cover failed in the compression zone when the beam reached its maximum load-carrying capacity for both conventional and GGBS-based RC beams without fibers. The crack depth is greater for fibre-reinforced GGBS-based RC beams compared to the respective control beams. Due to the fibre-bridging action, crack propagation is slow in fibre-reinforced beams. In addition, new cracks were formed and the widths of the existing cracks continued to widen in the case of the GGBS based beams with and without fibers. More cracks were observed in the fibre-reinforced concrete beams than in the beams without fibers. Especially in this regard, the width of the crack is greater in beams without fibres than in fiber-reinforced beams. Steel fibres played a significant role in reducing crack propagation and redistributing stress in fiber-reinforced beams, allowing more cracks to form in fiber-reinforced beams compared to beams without fibers. Figure 5a–h showed failure patterns of GGBS-based R C beams with and without fibers.

### Experimentation outcomes

The load carrying capacity of GGBS-based concrete beams without fibres is increased up to 60% replacement of cement by GGBS and is found to be higher than conventional concrete beams. This is due to a better bond and interlocking between the aggregates and the cement matrix. Further, the load-carrying capacity of the GGBS concrete beam with 80% cement replacement by GGBS is reduced compared to conventional concrete. A similar trend is observed for the long-term performance of RC beams composed of GGBS and fibers. The load-carrying capacity of the conventional concrete beam is 140 kN at the age of 28 days. At 28 days, the load-carrying capacity of GGBS concrete beams is 160 kN, 166 kN, 140 kN, and 110 kN for beams where the cement is replaced by 20%, 40%, 60%, and 80% of GGBS, respectively (Table 6). The load-carrying capacity of the control beam is 151 kN at 180 days, and for the GGBS concrete beams made up of cement replaced by 20%, 40%, 60%, and 80% with GGBS is 178 kN, 186 kN, 153 kN, and 116 kN, respectively. The load-carrying capacity is increased by up to 60% through the replacement of cement with GGBS due to the formation of additional C–S–H gel.

**Table 6:** Results of flexural performance of GGBS-based R C beams.

Mix ID	Mix designation	Load (kN) at 28 days		Ultimate deflection (mm)	Load (kN) at 180 days		Ultimate deflection (mm)
		First crack	Ultimate		First crack	Ultimate	
M9	C 450-0.5-G-00%, V_f_-0%	52	140	5.62	55	151	7.6
M9-1	C 450-0.5-G-20%, V_f_-0%	74	160	6.25	79	178	8.4
M9-2	C 450-0.5-G-40%, V_f_-0%	76	166	6.4	82	186	9.2
M9-3	C 450-0.5-G-60%, V_f_-0%	51	140	6.51	56	153	9.7
M9-4	C 450-0.5-G-80%, V_f_-0%	43	110	6.76	45	116	9.9
M9 A	C 450-0.5-G-00%, V_f_-0.5%	54	152	8.18	63	169	9.32
M9-1A	C 450-0.5-G-20%, V_f_-0.5%	81	180	8.84	84	189	11.26
M9-2A	C 450-0.5-G-40%, V_f_-0.5%	84	175	8.77	84	190	11.12
M9-3A	C 450-0.5-G-60%, V_f_-0.5%	58	150	6.9	60	162	9.21
M9-4A	C 450–0.5-G-80%, V_f_-0.5%	42	115	6.27	45	124	8.35
M9 B	C 450-0.5-G-00%, V_f_-1%	68	171	9.2	71	195	9.83
M9-1B	C 450-0.5-G-20%, V_f_-1%	79	210	9.6	82	227	10.62
M9-2B	C 450-0.5-G-40%, V_f_-1%	85	220	10.21	88	228	10.58
M9-3B	C 450-0.5-G-60%, V_f_-1%	73	172	8.5	82	175	8.4
M9-4B	C 450-0.5-G-80%, V_f_-1%	55	121	7.22	61	132	7.63
M9 C	C 450-0.5-G-00%, V_f_-1.5%	71	182	8.45	90	205	9.75
M9-1C	C 450-0.5-G-20%, V_f_-1.5%	90	212	9.75	95	233	10.38
M9-2C	C 450-0.5-G-40%, V_f_-1.5%	86	224	9.23	94	235	10.12
M9-3C	C 450-0.5-G-60%, V_f_-1.5%	75	180	8.54	86	187	8.92
M9-4C	C 450-0.5-G-80%, V_f_-1.5%	52	134	6.2	65	145	8.63

The test results proved that the load-carrying capacity of beams increased with the volume fraction of fibers ranging from 0.5 to 1.5% (reinforcing index 0.3–0.9). The load-carrying capacity of conventional concrete with a volume fraction of 0.5% fibers is 152 kN. The load-carrying capacity of GGBS concrete beams increased by about 15% at 28 days when the cement was partially replaced with GGBS up to 40%. And in the case of 60% replacement of cement with GGBS, the load-carrying capacity of GGBS-based concrete beam is nearly equal to that of conventional concrete. The load-carrying capacity is reduced by approximately 24% at 28 days when the cement was partially replaced with 80% of GGBS. The load-carrying capacity of conventional concrete with a volume fraction of 1% fibers is 171 kN. Moreover, as observed from the experimental results, the load-carrying capacity increased by about 28% when the cement was partially replaced with 40% GGBS and were added 1% of fibers were to the mix. The load-carrying capacity of the beam is reduced by approximately 36% at 28 days when cement was partially replaced by 80% of GGBS and adding 1% of fibers. The load-carrying capacity of conventional concrete is 182 kN when they are manufactured with 1.5% of the volume of fibers. Furthermore, observed that experimentally the load-carrying capacity of the GGBS concrete beam increased by about 23% when the cement was partially replaced with 40% GGBS, and the load-carrying capacity of the beam was nearly equal to conventional concrete when cement was replaced with 60% GGBS. There is a delay observed in the formation of the first crack when the cement was partially replaced with GGBS up to 60%. Table 6 consolidates various responses (first crack load, ultimate load, and ultimate deflection at 28 days and 180 days) of GGBS-based RC beams with and without fibers. From Fig. 5, it can be clearly noticed that (i) many flexural cracks were developed in the beams without fibers; (ii) many multiple cracks were developed in the beams containing fibers, indicating that more energy absorption and improved ductility; and (iii) the combination of flexure and shear cracks can also be seen in many beams.

### Load–deflection curves

**Figure 6:**
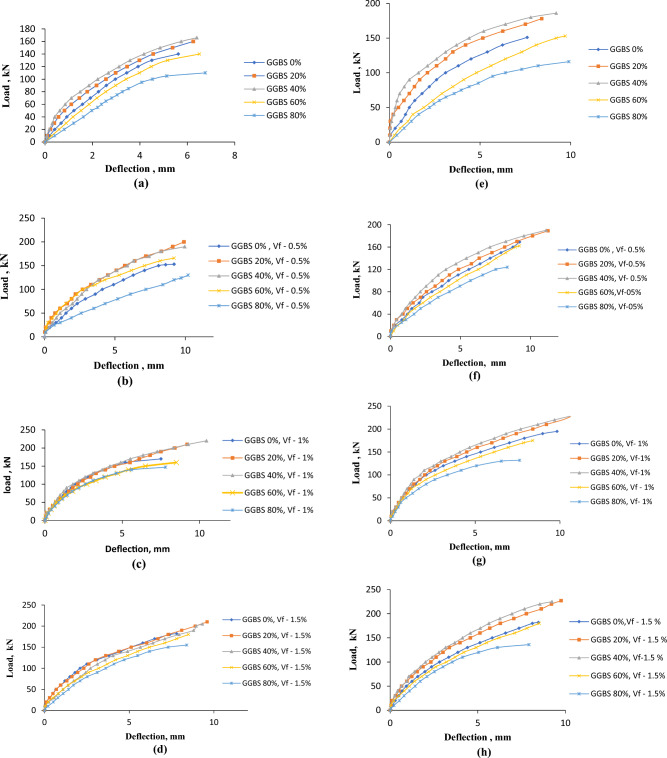
Load–deflection curves of RC beams. (**a**) Load–deflection curve of GGBS-based RC beam without fibers at 28 days. (**b**) Load–deflection curve of GGBS-based RC beam with the volume of fibers 0.5% at 28 days. (**c**) Load–deflection curve for GGBS-based RC beam with the volume of fibers 1% at 28 days. (**d**) Load–deflection curve for GGBS-based RC beam with the volume of fibers 1.5% at 28 days. (**e**) Load–deflection curve for GGBS-based RC beam without fibers at 180 days. (**f**) Load–deflection curve for GGBS-based RC beam with the volume of fibers 0.5% at 180 days. (**g**) Load–deflection curve for GGBS-based RC beam with the volume of fibers 1% at 180 days. (**h**) Load–deflection curve for GGBS-based RC beam with the volume of fibers 1.5% at 180 days.

Load–deflection curves are plotted by using the results obtained from the test. Since all the tests were conducted under load control, the responses were captured up to peak load (ultimate load) only. The load versus deflection of the control beam and the GGBS-incorporated beams (20–80%) with and without fibers at 28 and 180 days is shown in Fig. 6. The maximum load and associated deflection for the control beam are 140 kN and 5.62 mm, respectively, as shown in Fig. 6a and Table 6. In the case where GGBS replaces cement by 20%, 40%, 60%, and 80%, respectively, the load and associated deflections are 160 kN, 166 kN, 140 and 110 kN, 6.25 mm, 6.4 mm, 6.51 mm, and 6.76 mm. It can be clearly observed that the peak load is realised in the case of 40% replacement. Figure 6b–d exhibit the load–deflection behaviour of GGBS-based concrete beams containing 0.5%, 1%, and 1.5% steel fibres during a curing period of 28 days, whereas Fig. 6e–h depict the load–deflection curves at a curing period of 180 days. Additionally, it can be found that the load-bearing capacity of concrete beams made with GGBS is around 10–15% higher than that of control beams made without fibres. This investigation has an impact on all other cases, including the RC beams manufactured with GGBS and having steel fibre percentages of 1.0 and 1.5. From Table 6, it can also be observed that the first crack load, the ultimate load and the deflection corresponding to the ultimate load increase significantly with increasing the volume fraction of fibers. In general, the first crack load occurred at about 30–35% of the ultimate load of the corresponding beam.

### Development of analytical models

Analytical solutions will always be useful in predicting the response of structures and components. Several models have been proposed in the literature to predict the load–deflection behaviour of reinforced concrete beams with and without fibers. Equations are available in several codes of practice to predict theload–deflection of RC beams (Euro code, Canadian code, American code, and IS code). Several researchers have proposed expressions to predict the effective moment of inertia^22–24^. The general expressions are given below. The modular ratio, n is given by

1 $${\text{n}} = \frac{{{\text{E}}_{{\text{s}}} }}{{{\text{E}}_{{\text{c}}} }}.$$

where E_s_ is Young’s modulus of steel and E_c_, refers to Young’s modulus of concrete.

2 $$A_{st} = n*\pi r^{2} .$$

where r = radius of main steel reinforcement. The area of steel reinforcement (A_st_) in RC members is useful in determining the cracked moment of inertia of the section (I_cr),_

The centroid of the cracked section (y), which is a function of the percentage of tension reinforcement (p), breadth of the beam (b), aneffective depth of the beam (d) can be determined as.

3 $${\text{y}} = {\text{d}}\left( {\left( {\sqrt {\left( {{\text{np}}} \right)^{2} } + 2{\text{np}}} \right) - {\text{np}}} \right).$$

where p = A_st_/bd

Cracked moment of inertia of transformed section (I_cr_) can be obtained as follows:

4 $$I_{cr} = \left( {\frac{{{\text{by}}^{3} }}{3}} \right) + ({\text{nA}}_{{{\text{st}}}} \left( {{\text{d}} - {\text{y}})^{2} { }} \right)$$

The flexural deflection (Δ) for a simply supported RC beam of span L subjected to four-point bending load can be calculated by using the following equation

5 $$\Delta = \frac{{{\text{Pa}}}}{{48{\text{E}}_{{\text{c}}} {\text{I}}_{{\text{e}}} }}\left( {3{\text{L}}^{2} - 4{\text{a}}^{2} } \right)$$

The effective span of the beam (L) is 1000 mm, and the loading distance (a) is 330 mm. P = total applied load divided into two concentrated loads; Ec = modulus of elasticity of concrete; and Ie = effective moment of inertia of the beam cross section. After cracking (Ma ≥ Mcr), the building code ACI 318–08 [American Concrete Institute (ACI) 2008]^25^ and the Canadian concrete design standard CSA A23.3-04 [Canadian Standard Association (CSA) 2004]^26^, as well as the Standard Association of Australia (SAA 2001)^27^, use average effective moment of inertia (I_e_) to estimate the deflection of flexural members.

Young’s modulus of concrete (E_c_) from different codes such as ACI 318–08 and the Canadian concrete design standard CSA A23.3-04, European Design of Concrete Structures-Part 1(EN 1992-1-1: Euro code 2 (1992))^28^ and Indian standard Plain and reinforced concrete (IS 456:2000)^29^ can be used as per Table 7. The generalized formula for the determination of the effective moment of inertia (I_e_) is given below^30^.

6 $${\text{I}}_{{\text{e}}} = \left( {\frac{{{\text{M}}_{{{\text{cr}}}} }}{{{\text{M}}_{{\text{a}}} }}} \right)^{3} {\text{I}}_{{\text{g}}} + \left[ {1 - \left( {\frac{{{\text{M}}_{{{\text{cr}}}} }}{{{\text{M}}_{{\text{a}}} }}} \right)^{3} } \right]{\text{I}}_{{{\text{cr}}}} .$$

**Table 7:** Equations for Young’s modulus and rupture modulus by using various codes of practice.

S.NO	Description	AMERICAN CODE	CANADIAN	EURO CODE	IS 456 CODE
1	Young’s modulus of concrete	$E_{{\text{C}}} = 4700\sqrt {{\text{f}}_{{{\text{ck}}}} }$	${\text{E}}_{{\text{C}}} = 4500\sqrt {{\text{f}}_{{{\text{ck}}}} }$	${\text{E}}_{{\text{C}}} = \left( {22\left( {\frac{{{\text{f}}_{{{\text{ck}}}} }}{{10}}} \right)^{{0.3}} } \right)*1000$	${\text{E}}_{{\text{C}}} = 5000\sqrt {{\text{f}}_{{{\text{ck}}}} }$
2	Modulus of rupture of concrete	${\text{f}}_{{\text{r}}} = 0.62\sqrt {{\text{f}}_{{{\text{ck}}}} }$	$f_{{\text{r}}} = 0.6\sqrt {{\text{f}}_{{{\text{ck}}}} }$	${\text{f}}_{{\text{r}}} = 0.3({\text{f}}_{{{\text{ck}}}} )^{2/3}$	${\text{f}}_{{\text{r}}} = 0.7\sqrt {{\text{f}}_{{{\text{ck}}}} }$

According to this equation, with increasing the load on the beam, I_e_ gradually decreases from I_g_ to I_cr_, depending on the ratio of the cracking moment M_cr_ to the applied moment M_a_. This equation was especially deloped for determining the deflection of RC beams with low reinforcement ratios or FRP reinforced concrete beams. But it is the basic form of the equation which helps us in determining the effective moment of inertia equation for flexural behaviour of GGBS-based cement concrete^31^. The following equation is used in the present study for the determination of I_e_^28^.

7 $${\text{I}}_{{\text{e}}} = \propto_{{\text{t}}} {\text{I}}_{{{\text{cr}}}} \left( {\frac{{{\text{Mcr}}}}{{{\text{Ma}}}}} \right)^{0.4} .$$

From the Eq. (7), the effective moment of inertia Ie depends on the ratio of the M_cr_/M_a_.

**Figure 7:**
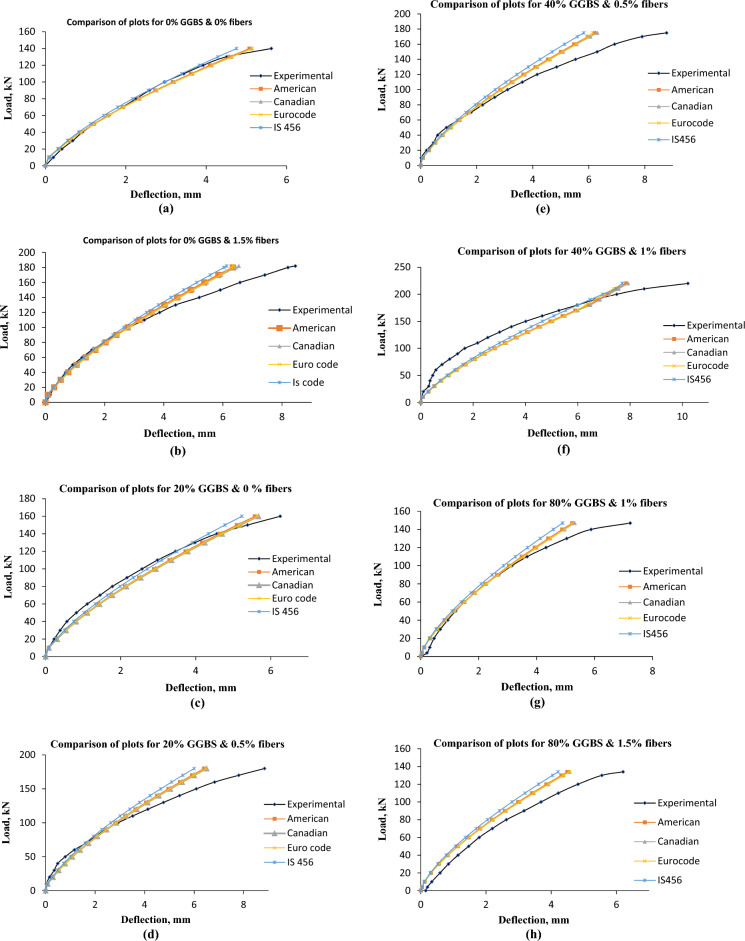
Comparison Plots about RC beam load–deflection curves. (**a**) Load–deflection curve for conventional R C beam with 0% GGBS and the volume of fibers 0%. (**b**) Load–deflection curve for conventional R C beam with 0% GGBS and the volume of fibers 1.5%. (**c**) Load–deflection curve for conventional R C beam with 20% GGBS and the volume of fibers 0%. (**d**) Load–deflection curve of conventional R C beam with 20% GGBS and the volume of fibers 0.5%. (**e**) Load–deflection curve for conventional R C beam with 40% GGBS and the volume of fibers 0.5%. (**f**) Load–deflection curve for conventional R C beam with 40% GGBS and the volume of fibers 1%. (**g**) Load–deflection curve for conventional R C beam with 80% GGBS and the volume of fibers 1%. (**h**) Load–deflection curve for conventional R C beam with 80% GGBS and the volume of fibers 1.5%.

$\propto_{{\text{t}}}$. is the term, plays a major role in determining (I_e_), which further depends on the constant (${\upbeta }_{{\text{t}}}$.).

8 $$\propto_{{\text{t}}} = 0.65{\upbeta }_{{\text{t}}} + 1.76$$

where β_t_ = 1.2 is an assumed value. Where M_cr_ = Cracked moment of inertia and M_a_ refers to applied moment.

9 $$\left( {{\text{M}}_{{\text{a}}} = \frac{{{\text{Pa}}}}{2}} \right)$$

where M_a_ = Actual moment due to load, where P = Load acting on the beam at the distance ‘a’ from the support.

10 $${\text{M}}_{{{\text{cr}}}} = \left( {\frac{{{\text{f}}_{{\text{r}}} {\text{*I}}_{{\text{g}}} }}{{{\text{Y}}_{{\text{t}}} }}} \right).$$

11 $${\text{I}}_{{\text{g}}} = \frac{{{\text{bd}}^{3} }}{12}$$

where f_r_ = modulus of rupture, which is the maximum bending stress that can be applied to that material before it yields. The gross moment of inertia (I_g_) is the resistance offered by the section to its rotation, and Y_t_is the centroid of the gross section.

using the above modified equations for effective moment of inertia, cracked moment of inertia, and several important parameters such as modulus of rupture, Young’s modulus of concrete (ACI code, Canadian code, Euro code, and IS 456 code as per Table 7), deflections were determined for various RC beams and compared with the corresponding experimental observations. Figure 7a–h present the load–deflection comparison plot for various RC beams with and without GGBS and % of fibers.From Fig 7a, it can be observed that the experimental maximum load-carrying capacity of the RC beam of 0% GGBS and 0% fibres is 140 kN and the corresponding peak deflection is 5.62 mm for the experimental result. The deflection obtained by using American, Canadian, Euro-code, and IS 456 codes is 5.08 mm, 5.13 mm, 5.15 mm, and 4.76 mm, respectively^25,26,28,29^. The deflection obtained by using American, Canadian, and Euro-code is in good agreement with the experimental value. The deflection obtained by using the IS code differs from the experimental value. According to Fig. 7b, the RC beam with 1.5% fibres and 0% GGBS has an experimental maximum load-bearing capacity of 182kN and a peak deflection of 8.45 mm. Using the American, Canadian, Euro-code, and IS 456 codes, the resulting deflection is 6.43 mm, 6.54 mm, 6.36 mm, and 6.13 mm, respectively. As shown in Fig. 7c has an experimental maximum load-bearing capacity of 160 kN and a peak deflection of 6.25 mm for the RC beam with 20% GGBS and 0% fibres. The resulting deflection, using the American, Canadian, Euro-code, and IS 456 codes, is 5.58 mm, 5.67 mm, 5.64 mm, and 5.23 mm, respectively. The RC beam with 20% GGBS and 0.5% fibres exhibits a peak deflection of 8.84 mm and an experimental maximum load-bearing capacity of 180 kN, as shown in Fig. 7d. The resulting deflection, using the American, Canadian, Euro-code, and IS 456 codes, is 6.40 mm, 6.50 mm, 6.47 mm, and 6.00 mm, respectively.

According to testing results, the RC beam with 40% GGBS and 0.5% fibres has a maximum load bearing capacity of 175kN and a peak deflection of 8.76 mm, as illustrated in Fig. 7e. The deflection measurements obtained using the American, Canadian, Euro-code, and IS 456 codes were 6.20 mm, 6.31 mm, 6.14 mm, and 5.82 mm, respectively. The experimental maximum load bearing capacity of the RC beam made up of 40% GGBS and 1% fibres is 220 kN, and its peak deflection is 10.21 mm, as shown in Fig. 7f. The resulting deflection is 7.84 mm, 7.93 mm, 7.70 mm, and 7.72 mm when using the American, Canadian, Euro-code, and IS 456 codes, respectively. The RC beam with an 80% GGBS and 1% fibre composition shown in Fig. 7g has an experimental maximum load bearing capacity of 147 kN and a peak deflection of 7.22 mm. The resulting deflection, using the American, Canadian, Euro-code, and IS 456 codes, is 5.22 mm, 5.31 mm, 5.23 mm, and 4.90 mm, respectively. The peak deflection as a result of the experimental maximum load bearing capability of the RC beam made up of 80% GGBS and 1.5% fibres is 6.20 mm, as illustrated in Fig. 7h. Using the American, Canadian, Euro-code, and IS 456 codes, the resulting deflection is 4.49 mm, 4.57 mm, 4.55 mm, and 4.21 mm, respectively. In light of the discussion above, it is clear that (i) the deflections obtained using the American, Canadian, and Euro-code are closer to experimental observations, and (ii) the differences in the predicted and the corresponding experimental deflection are mainly due to the expressions used for computation of Young’s modulus relations and effective moment of inertia parameters.

### Moment curvature relationship of RC beams

**Figure 8:**
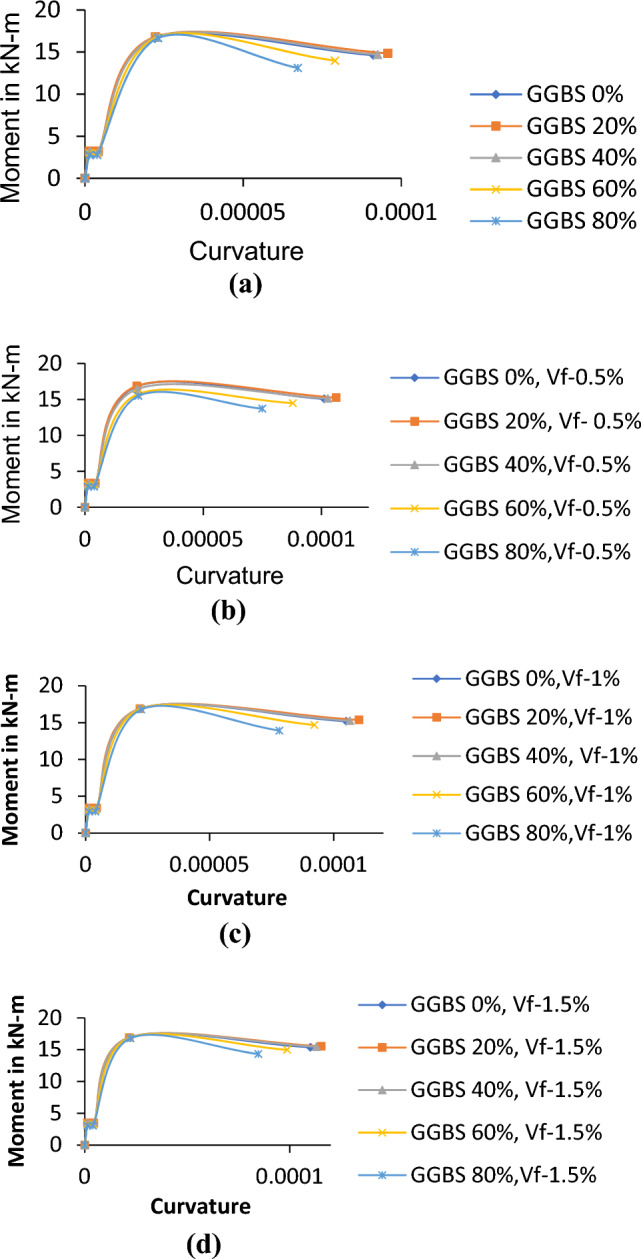
Moment curvature relationship of RC beams. (**a**) Moment curvature relationship of GGBS concrete without fibers. (**b**) Moment curvature relationship for GGBS concrete with the volume of fibers 0.5%. (**c**) Moment curvature relationship for GGBS concrete with the volume of fibers 1.0%. (**d**) Moment curvature relationship for GGBS concrete with the volume of fibers 1.5%

For this purpose, consider the specimens having the same area of longitudinal and shear reinforcements but different percentage replacements of GGBS and volume of fibers. To deduce the moment–curvature curve, the forces were determined in the section step by step with an increase in strain. After establishing the tensile and compressive balance in the section, the total moment and curvature were obtained in each step. The Moment–Curvature relations (Fig. 8a–d) were developed for 28 days strength because, in the design of structural elements, the strength of concrete can be taken as a characteristic strength of concrete corresponding to 28 days strength. In the uncracked portion, lesser curvature can be seen in the case of the concrete mixes exhibiting lesser strength. It is observed to be more for high-strength concrete mixes because the flexural rigidity of concrete increases with strength. Stiffness variation can also be seen as similar to curvature^24,30,32^. In addition to that, the moment carrying capacity of GGBS based RC beams is higher than conventional RC beams and also the ultimate moment carrying capacity and the curvature of the beam increase by incorporating fibers in different volume fractions and also compared with beams without fibers.

## Summary and conclusions

The following is the summary and conclusions from the experimental and analytical investigation of GGBS-based RC beams with and without fibres:

- The load carrying capacity of the conventional concrete beam (140 kN) is found to be increased for the cement is partially replaced with GGBS up to 40% and equals the load carrying capacity when compared with GGBS of 60%. The increase in load is due to better bond and interlocking between the aggregates and the cement matrix, but for 80% GGBS, there is a reduction in the load-carrying capacity (110 kN) compared to the control beam. A similar trend is observed for the long-term performance of RC beams composed of GGBS and fibers.
- The load-carrying capacity of conventional concrete with a volume fraction of 0.5% fibers, 1% fibers and 1.5% fibers is 152 kN, 171 kN, 182 kN, respectively and with GGBS, the percentage increase in load-carrying capacity is 15%, 28%, and 23% respectively for the corresponding volume of fibres up to 40% GGBS replacement. Similarly, the deflection values of controlled beams are lower compared to the beams having higher GGBS percentages. It was clearly observed that the peak load is realized for the case of 40% replacement. Since the experiments were conducted under load control, deflections cannot be compared.
- It was primarily noticed that (i) many flexural cracks were developed in the beams without fibers; (ii) many multiple cracks were developed in the beams containing fibers, indicating more energy absorption and improved ductility; and (iii) the combination of flexure and shear cracks were also seen in many beams.
- The crack depth is found to be higher for fibre-reinforced GGBS-based RC beams compared to the respective control beams. Due to the fibre-bridging action, crack propagation is slow in fibre-reinforced beams. In addition, new cracks were formed and the widths of the existing cracks continued to widen in the case of the GGBS-based beams with and without fibers. More cracks were observed in the fibre-reinforced concrete beams than in the beams without fibers. The width of the crack is greater in beams without fibres than in fiber-reinforced beams. Steel fibres played a significant role in reducing crack propagation and redistributing stress in fibre-reinforced beams, allowing more cracks to form in fibre-reinforced beams compared to beams without fibers.
- Analytical deflection is predicted by using various Codes of practice, namely, American, Canadian, Euro-code and IS456 codes. It was majorly observed that the deflection is strongly dependent on the effective moment of inertia, cracked moment of inertia, modulus of rupture, Young’s modulus of concrete etc.
- The deflections obtained by using the above Codes of practice have been compared with the corresponding experimental observations. From the overall study, it can be summarized that the deflections obtained by using American, Canadian, and Euro-code are closer to experimental observations. The models could be improved by accounting for stiffening and bond aspects to predict the deflections close to the experimental findings.
- From the experimental findings, it was observed that by the addition of fibers to the concrete, moment carrying capacity and curvature increase compared to the GGBS beam without fibers. The curvature corresponding to the moment was found to increase for the high-strength members. The beam possessing high strength exhibited more stiffness than lower-strength beams.

## Data Availability

The datasets used and/or analysed during the current study available from the corresponding author on reasonable request.
